# Influence of reflective foil on persimmon (*Diospyros kaki* Thunb.) fruit peel colour and selected bioactive compounds

**DOI:** 10.1038/s41598-019-55735-1

**Published:** 2019-12-13

**Authors:** Tina Smrke, Martina Persic, Robert Veberic, Helena Sircelj, Jerneja Jakopic

**Affiliations:** 1 University of Ljubljana, Biotechnical Faculty, Department of Agronomy, Chair for Fruit Growing, Viticulture and Vegetable Growing, Jamnikarjeva 101, SI-1000 Ljubljana, Slovenia; 2 University of Ljubljana, Biotechnical Faculty, Department of Agronomy, Chair of Applied Botany, Plant Ecology & Physiology and Informatics, Jamnikarjeva 101, SI-1000 Ljubljana, Slovenia

**Keywords:** Light responses, Secondary metabolism, Environmental impact

## Abstract

The purpose of this work was to investigate how to overcome the negative effect of anti-hail netting on the photosynthetic photon flux density (PPFD) in persimmon trees and persimmon fruit colour, flesh firmness, total soluble solids (TSS) and individual carotenoid and phenolic compound contents (determined via HPLC-MS) under a hail net with the use of reflective foil. Reflective foil increased the PPFD on the lower side of the fruits, while there was no significant difference on the upper side compared to those of the control group. The CIE colour parameters a* and *h°* indicated more intense red colouration of the fruits in the foil treatment than those in the control. Among carotenoids, the content of β-carotene increased, and the content of zeaxanthin decreased in fruits in the reflective foil treatment group, while the content of other carotenoids was not affected by the reflective foil. Among individual phenolic compounds in the persimmon peel, greater light intensity significantly influenced all three phenolic compound subgroups: phenolic acids, flavan-3-ols and flavonols. The content of gallic acid in the persimmon flesh increased the most, while other phenolics did not show any significant differences in concentrations between the foil and control groups. This study is the first to examine the influence of reflective foil on bioactive compounds in persimmon fruit. The use of reflective foil in persimmon orchards improves persimmon fruit colour and selected bioactive compound contents.

## Introduction

Persimmon (*Diospyros kaki* Thunb.) is a widespread fruit, with very attractive yellow to orange or dark red peel. The dark red peel colour is a key characteristic that makes the fruit attractive to the consumer^1^. It is believed that a key regulating factor that alters persimmon fruit colour is light^2^. The persimmon canopy is very dense, and together with large leaves, it makes it difficult for light to penetrate through the canopy, resulting in shading of the fruits, especially those positioned in lower parts of the tree, which leads to poor colouration of the fruits^3^.

The frequent occurrence of hailstorms, in part due to global warming^4^, has led fruit growers to introduce hail nets in their orchards, despite the additional investment^5^. Mupambi *et al*.^6^ reported that hail damage to leaves reduces overall photosynthesis and affects fruit quality. Nets are also used for protection against pests, wind and excessive solar radiation damage. The major disadvantage of protective nets is the interception of photosynthetic photon flux density (PPFD), which is important for plant growth. Consequently, with optimum nutrient and water supply, light becomes a limiting factor for optimal yield quality and quantity in intensive persimmon production^7^.

Persimmon is a climacteric fruit that, in addition to primary metabolites, minerals and vitamins, is also rich in phenolic compounds, carotenoids and other bioactive compounds. The sugar content in persimmon flesh is higher than in apples, peaches, oranges and pears, amounting to 12.5 g/100 g fresh weight. The most common sugars are glucose and fructose^8^.

Much research with promising results has been conducted on light intensity improvement in the intensive production of various fruits, including apples^9,10^, pears^11^, cherries^12^, persimmon^7^ and kiwifruit^7^, by covering the orchard floor with reflective foil (RF). This enables light reflection from the ground into the inter-row zone and tree canopy, thus improving light intensity and distribution in the dense persimmon canopy, as mentioned above. Consequently, fruits positioned in the shaded area intercept reflected light, which led to improved fruit colouration^10^. Increased levels of PPFD in the canopy may improve fruit quality, such as improved peel colouration^10,13^.

The aim of our study was to evaluate the influence of reflective foil on light intensity in persimmon trees, persimmon peel colour, total soluble solids (TSS), fruit firmness, content of carotenoids in persimmon fruit and content of phenolic compounds in fruit peel and flesh in the ‘Tipo’ variety. The ‘Tipo’ variety was chosen for this experiment because it is a popular cultivar and the most appropriate one for climate conditions in Slovenia.

## Materials and Methods

### Plant material

The experiment was carried out on 9-year-old ‘Tipo’ persimmon trees, grown under a hail net, in the test field of the Biotechnical Faculty, located in Orehovlje (latitude: 45°89′N, longitude: 13°63′E, altitude: 48 m) in western Slovenia, one month before harvest time. Reflective foil (RF) was placed on the ground on 10 October 2018; the control (C) treatment was without foil. Measurements were carried out on five individual trees for each treatment, with uniform fruit load and canopy density. Three uniform, healthy fruits, without injuries, at a similar exposition in the canopy of each tree and at a height of one to two metres, were visually selected and labelled on each tree for light and peel colour measurements. Six fruits per tree were harvested on 8 November, of which three of them were the abovementioned labelled fruits. They were transported to the laboratory, where fruit colour, flesh firmness and total soluble solids (TSS) were measured. Immediately after the measurements, ten fruits per treatment were cut into pieces, frozen in liquid nitrogen, lyophilized and stored at −80 °C for carotenoid extraction. Approximately half of the fruits were peeled, and the fruit flesh and peel were separately frozen in liquid nitrogen, lyophilized and stored at −20 °C until phenolic compound extraction, which was performed in ten replicates.

### Light measurements

PPFD was measured in the orchard and on each labelled fruit. On each marked fruit, fifteen for each treatment, PPFD was measured on the upper and lower parts of the fruit using a Li-Cor quantum sensor (µmol m^−2^ s^−1^). Light was also measured under the net in each treatment, on the ground and at a height of 1 metre between the rows, using a 1 metre long sensor (Li-Cor; µmol m^−2^ s^−1^), with the sensor facing upward. At the same time, we measured the light outside the net, also at two different heights. For each variable, five repetitions of measurements were made. The light was measured on a clear, sunny day.

### Fruit colour, flesh firmness and total soluble solids (TSS) measurements

Persimmon fruit peel colour was measured on six fruits per tree, using a Konica Minolta CR-10 Chroma portable colorimeter, one month before harvest and at harvest time. Colour was measured at the same point on the fruit equator for both measurements. The most common scale for determining fruit colour was developed by the Commission Internationale d’Eclairageusing (CIELAB), whereby each colour represents an exact point in a three-dimensional system. The parameters used to describe colour are L*, h°, a*, b* and C*. The L* value represents lightness on a dark-bright scale from 0 to 100, where 0 is black and 100 is white. The hue angel (*h°*) represents colour expressed in degrees, from 0° to 360°, where 0°–90° is red towards yellow, 90°–180° is yellow towards green, 180°–270° is green towards blue and 270°–360° is blue towards red. Parameters a* and b* correspond to values from −128 to 127, where a negative value of a* represents green and positive represents red, and a negative value of b* represents blue and positive represents yellow. The C* value corresponds to colourfulness; that is, the higher the value, the more intense the colour.

Persimmon peel was removed on two sides of each marked fruit, and flesh firmness was measured twice using a digital penetrometer (TR, Turini, Italia; kg cm^−2^) with an 8 mm diameter tip.

Total soluble solids (TSS) were measured using a digital refractometer (Milwaukee, MA885) by squeezing the juice from the fruit onto the measuring point. TSS content is expressed in °Brix.

### Analysis and determination of individual carotenoids

Extraction of carotenoids occurred under reduced light and low temperature. Each sample was ground separately in liquid nitrogen with a mortar and pestle into a fine powder, and 0.5 g of sample was mixed with 3 mL of hexane:acetone:ethanol (50:25:25; v-v:v)^14^ and put into a cooled ultrasonic bath for one hour. After extraction, samples were centrifuged for 8 min at 4 °C and 10 000 rpm. The supernatant was then filtered through Chromafil AO-20/25 polyamide filters (Macherey-Nagel, Germany; pore size: 0.2 µm) into vials.

Individual carotenoids were analysed based on the method previously described by Sircelj and Batic^15^, with some modifications, using the Dionex UltiMate 3000 high-performance liquid chromatography (HPLC; Thermo Scientific) system equipped with a diode array detector (DAD) at 450 nm and a Gemini C18 column (150 × 4.6 mm 3 µm, Phenomenex) at 25 °C. The flow rate was maintained at 0.6 mL min^−1^, and the auto sampler (Vanquish) temperature was maintained at 10 °C. The injection volume was 20 µL. Mobile phase A was acetonitrile with methanol and double distilled water (100/10/5; v/v/v), and mobile phase B was acetone and ethyl acetate (2/1; v/v). The linear gradient was from 10% solvent B to 70% solvent B in the first 18 min, then a linear flow of 70% B to 24 min and back to the initial conditions to the end of the run. The run time was 40 min. Carotenoids were identified by comparing the retention times of the analytes with those of external standards. The content of individual carotenoids was calculated from corresponding external standards and expressed in mg g^−1^ of dry weight (DW).

### Analysis and determination of individual phenolic compounds

Extraction of individual soluble phenolic compounds was carried out in accordance with the method previously described by Mikulic-Petkovsek *et al*.^16^ For extraction, 1 g of lyophilized material (fruit peel or flesh) was mixed with 5 mL of 80% methanol containing 3% formic acid. The samples were homogenized by vortexing and extracted in an ultrasonic bath for one hour. Extracts were then centrifuged at 10 000 rpm and 4 °C for 12 min, and the supernatant was filtered through 0.2 µm Chromafil AO-20/25 polyamide filters (Macherey-Nagel, Germany) into vials.

Soluble phenolic compounds were separated on a Dionex UltiMate 3000 HPLC (Thermo Scientific) system, and absorbance was monitored at 280 and 350 nm. The flow rate was 0.6 ml min^−1^, and the auto sampler temperature was 10 °C. The separation was performed on a Gemini C18 (150 × 4.6 mm 3 µm, Phenomenex) column at 25 °C. Mobile phase A was 3% acetonitrile with 0.1% formic acid in double distilled water (v/v/v), and mobile phase B was 3% double distilled water with 0.1% formic acid in acetonitrile (v/v/v). The following linear gradient was used: 0–15 min, 5% solvent B; 15–20 min, 20% B; 20–30 min, 30% B; 30–35 min, 90% B; and 35–45 min, 100% B before returning to the initial conditions to the end of the run time, which was 50 min. The injection volume was 20 µL.

Identification of individual phenolic compounds was performed with an LTQ XL mass spectrometer (Thermo Scientific) operating in negative ion mode with electrospray ionization (ESI). The parameters for the analysis were as follows: the source voltage was 4 kV, the capillary temperature was 250 °C and the sheath and auxiliary gases were 20 and 8 units, respectively. The injection volume was 10 µL, and the flow rate was maintained at 0.6 ml min^−1^. *m/z* scanning was performed from 115 to 1600. The phenolic compounds were identified based on their mass fragmentation pattern and their retention times. Their content was calculated from standard curves and expressed as mg kg^−1^ of DW.

### Chemicals

The following standards were used to determine the carotenoid and phenolic compounds: lycopene, zeaxanthin, lutein, *β*-cryptoxanthin, *α*-carotene and *β*-carotene from DHI LAB Product (Hørsholm, Denmark), neochlorogenic acid from Sigma-Aldrich Chemie (Steinhein, Germany) and gallic acid, caffeic acid, *p*-coumaric acid, catechin, procyanidin B1, procyanidin dimer, quercetin-3-*O*-glucoside, quercetin-3-*O*-galactoside, quercetin-3-*O*-rutinoside and kaempferol-3-*O*-glucoside from Fluka Chemie (Steinhein, Germany).

Other chemicals used, including hexane, acetone, ethanol, methanol, acetonitrile, formic acid and ethyl acetate, were from Sigma-Aldrich. The water used for sample preparation, solutions and analyses was twice distilled and purified with a Milli-Q water purification system by Millipore (Bedford, MA).

### Statistical analyses

Statistical analysis was performed in R commander i386 3.5.2. Statistically significant differences between treatments were determined by one-way analysis of variance (ANOVA). Differences between treatments were estimated using the LSD test and were considered significant at *P* < 0.05. Significant differences between treatments are represented by different letters.

## Results and Discussion

### Effect on light

In some regions, the introduction of hail netting in persimmon orchards is necessary to ensure fruit quality^3^. A consequence is a smaller amount of light penetrating through the canopy, which could decrease the orange colouration of persimmon fruits. We measured the PPFD under a hail net and outside of it. Under the hail net at a 1 m height, the PPFD was 27–30% lower (929 µmol m^−2^ s^−1^) compared to that of the open field (1297 µmol m^−2^ s^−1^). Our results are consistent with the reports of other authors^9,10^.

On the ground, PPFD was 27–32% lower under the net (857 µmol m^−2^ s^−1^) compared to that measured outside the net (1217 µmol m^−2^ s^−1^). Persimmon fruit facing inside the canopy received less light than the part facing outside^7^. By introducing reflective foil in intensive persimmon production, growers try to improve the light distribution inside the canopy and expose lower and internal parts of the fruits to a greater amount of light^10^.

We measured the light on the upper and lower parts of the fruits (Table 1). There was no significant difference in PPFD on the upper part of the fruit, where we measured 36.8 µmol m^−2^ s^−1^ in the foil treatment and 36.4 µmol m^−2^ s^−1^ in the control treatment. In both cases, reduced light by the hail net was not able to penetrate through the dense persimmon canopy. The lowest amount of PPFD in the lower part of persimmon fruit was measured in the control (22.5 µmol m^−2^ s^−1^), while fruits in the foil treatment group (52.5 µmol m^−2^ s^−1^) had 233% higher PPFD than that of the control on the lower side. This can be explained by increased light reflection from the foil compared to the amount of light in the control group. Similar results are reported by Jakopic *et al*.^10^ on ‘Fuji’ apples.

**Table 1:** Lighting on the upper and lower parts of persimmon fruits in the control and reflective foil treatment groups one month before harvest (µmol m^−2^ s^−1^)

	Control	Reflective foil	Significance
Upper side	36.4 ± 2.1	36.8 ± 4.2	NS
Lower side	22.5 ± 2.0 a	52.5 ± 3.8 b	*

Average values ± standard errors of fifteen independent measurements are presented. Different letters in a row (a-b) represent statistically significant differences between treatments according to an LSD test at α < 0.05.

### Effect on fruit colour

Persimmon fruit colour depends on the light quantity and its distribution in the canopy and inter-row zones between the trees^2^. In our experiment, the persimmon fruit peel, which is orange in the ‘Tipo’ variety, had significantly lower *h*° in the reflective foil treatment group (66.0 ± 0.6) than in the control group (67.7 ± 0.6) (Table 2), which is, according to CIE, closer to red. The more orange colouration of fruits above the foil could be attributed to the direct exposure of reflected light in comparison with the exposure in the control group^7^, in which the fruits had a *h*° closer to the yellow area of the scale. Our findings are consistent with the results of Thorp *et al*.^7^, who measured lower *h*° values in ‘Fuyu’ persimmon fruit when reflective foil was used.

**Table 2:** Persimmon peel colour, flesh firmness (kg m^−2^) and TSS measurements (°Brix) in the control and reflective foil treatment groups.

	Control	Reflective foil	Significance
*h°*	67.7 ± 0.6 b	66.0 ± 0.6 a	*
a*	23.9 ± 0.7 a	26.2 ± 0.6 b	*
b*	57.9 ± 0.8	58.9 ± 1.0	NS
L*	64.3 ± 0.3	64.1 ± 0.2	NS
Flesh firmness (kg cm^−2^)	2.23 ± 0.03	2.17 ± 0.03	NS
Total soluble solids (°Brix)	15.8 ± 0.18	15.9 ± 0.24	NS

Average values ± standard errors of fifteen independent peel colour parameter measurements, two flesh firmness (kg m^−2^) measurements per sample and one TSS (°Brix) measurement per sample are presented. Different letters in a row (a-b) represent statistically significant differences between treatments according to an LSD test at α < 0.05.

The use of reflective foil affected the CIE colour parameter a*. The values were significantly higher in the fruits of the reflective foil treatment group, which indicates a higher proportion of red colour compared to that of the fruits in the control. There was no significant difference in CIE colour parameters b* and L*. It can be concluded that the use of reflective foil caused more orange and less yellow fruit but did not affect fruit lightness. Thorp *et al*.^7^ reported an advanced harvest date by almost a week for persimmon fruits in the lower canopy zone because of better peel colouration.

### Effect on flesh firmness and total soluble solids (TSS)

Persimmon flesh firmness (kg cm^−2^) and total soluble solids (TSS; °Brix) were measured at harvest (Table 2). There was no significant difference between treatments in the two parameters, which leads to the conclusion that the reflective foil did not affect the maturation of fruits. However, some authors suggest that reflective foil can be a useful tool to accelerate the ripening process of persimmon fruit^7^. In contrast, Jakopic *et al*.^10^ reported an increase in the TSS content in apple flesh in the control group, while the used of foil caused a reduction of TSS.

### Effect on the content of individual carotenoids

Carotenoids are bioactive compounds that contribute to both persimmon fruit colour and nutritional value. They are important parts of the human daily diet, especially β-carotene, α-carotene and cryptoxanthins, as vitamin A precursors^17^. Their content in persimmon flesh and peel is similar, so the extraction was made from the whole fruit^18^.

**Figure 1:**
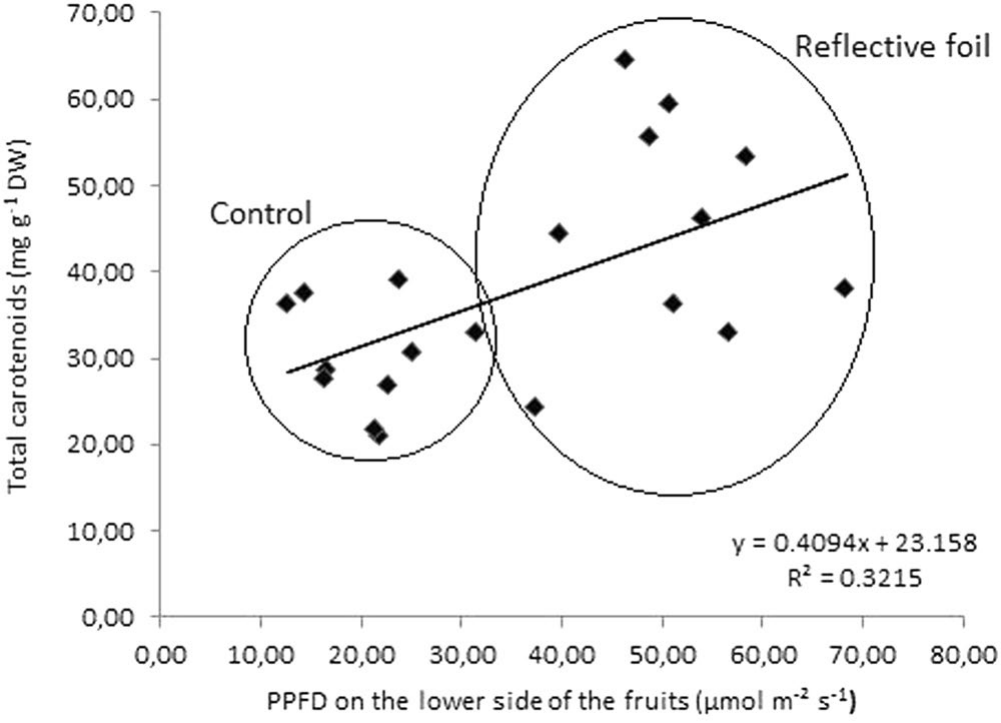
Relationship between total carotenoid content (mg g^−1^ DW) in persimmon fruits and PPFD on the lower side of the fruits (µmol m^−2^ s^−1^) in the control and reflective foil treatment groups.

Carotenoid synthesis is regulated by light. Figure 1 shows the relationship between carotenoid content in persimmon fruits and PPFD on the lower side of the fruits in the control and reflective foil groups. As the PPFD on the fruits increased, as a result of reflection from the foil, the carotenoid content in fruits also increased (p = 0.009). However, carotenoid synthesis is not only light dependent (R^2^ = 0.3215) but is also influenced by other factors, such as genetic background, maturity and other environmental conditions^18^.

Individual carotenoids were separated using HPLC and identified by comparing them to internal standards. The corresponding chromatogram are shown in Supplementary Fig. S1. The most abundant carotenoid in persimmon fruit in both treatment groups was β-carotene, which was significantly higher in the reflective foil treatment group (Table 3). This is in accordance with the reports of other authors, which already reported that β-carotene is the predominant carotenoid in persimmon fruit^19^. On the other hand, Zhou *et al*.^18^ reported that the highest content of an individual carotenoid in persimmon fruits was that of β-cryptoxanthin^18^.

**Table 3:** Individual carotenoids identified in persimmon fruit and their content in the control and reflective foil treatment groups (mg g^−1^ DW).

Individual carotenoid	Control	Reflective foil	Significance
Lutein	2.29 ± 0.14	2.26 ± 0.18	NS
Zeaxanthin	0.15 ± 0.02 b	0.09 ± 0.01 a	*
β-cryptoxanthin	0.61 ± 0.04	0.50 ± 0.03	NS
Lycopene	1.05 ± 0.04	1.03 ± 0.05	NS
α-carotene	0.51 ± 0.02	0.49 ± 0.01	NS
β-carotene	24.12 ± 1.78 a	42.57 ± 3.18 b	*

Average values ± standard errors of ten independent samples are presented. Different letters in a row (a-b) represent statistically significant differences between treatments according to an LSD test at α < 0.05.

An increased amount of PPFD is known to stimulate β-carotene synthesis, as clearly shown by Zhang *et al*.^17^. They applied direct light to Satsuma mandarin and Valencia orange juice sacs, with intensities of 50 and 100 µmol m^−2^ s^−1^, which is similar to the PPFD on the lower parts of persimmon fruits in the reflective foil group in our experiment.

Of the xanthophyll-cycle pigments, zeaxanthin has the most positive effect on human health since it prevents and treats certain eye diseases^20^. For that reason, high zeaxanthin content is desirable in persimmon fruits. The zeaxanthin content in our persimmon fruit samples was significantly higher in the control than in the foil treatment group, which contradicts the previously reported results^21^. However, Veberic *et al*.^19^ and Zhou *et al*.^18^ measured even lower contents of zeaxanthin in persimmon flesh and peel in the ‘Tipo’ variety and in 46 different varieties, respectively. This can be explained by carotenoid extraction from fresh fruit samples, where values are always lower compared to those extracted from lyophilised material^19^.

Other carotenoid contents in persimmon fruit did not change under the influence of enhanced light reflection. The lutein, β-cryptoxanthin, lycopene and α-carotene contents were slightly higher in the control group than in the foil treatment group, but the differences were not statistically significant. In contrast, Zhang *et al*.^17^ reported higher contents of lutein, β-cryptoxanthin and α-carotene in the Satsuma mandarin group and Valencia orange group juice sacs under light treatment than those in the control group. Different plants or species differ from each other in proportion to individual carotenoid content in their cells. This could be the reason for the different responses of plants to enhanced PPFD regarding individual carotenoid biosynthesis^21^.

### Effect on the content of individual phenolic compounds

Among phenolics, gallic acid, ferulic acid and *p*-coumaric acid are most represented in persimmon flesh^22^, which partially corresponds with our results (Table 4). Furthermore, dihydrocaffeic acid 3-glucuronide, caffeoylquinic acid, catechin and quercetin-3,4-diglucoside were identified in our samples of persimmon ‘Tipo’ flesh via comparison to standards and/or confirmed by ESI-MS (see Supplementary Figs. S2 and S3).

**Table 4:** Individual phenolic compounds and the sum of phenolic acids, flavan-3-ols and flavonols identified in persimmon flesh and their content in the control and reflective foil treatment groups (mg kg^−1^ DW).

Individual phenolic compound	Control	Reflective foil	Significance
Gallic acid	80.8 ± 2.8 a	102.7 ± 4.1 b	*
Dihydrocaffeic acid 3-glucuronide^A^	87.9 ± 4.0	82.3 ± 5.7	NS
Caffeoylquinic acid^B^	431.3 ± 19.4	403.7 ± 28.0	NS
*p*-Coumaric acid	0.578 ± 0.04	0.675 ± 0.05	NS
**Phenolic acids**	**600.6 **±** 26.2**	**589.4 **±** 37.9**	**NS**
Catechin	10.6 ± 1.6	11.4 ± 2.0	NS
**Flavan-3-ols**	**10.6 **±** 1.6**	**11.4 **±** 2.0**	**NS**
Quercetin-3,4-diglucoside	0.3 ± 0.02	0.3 ± 0.03	NS
**Flavonols**	**0.3 **±** 0.02**	**0.3 **±** 0.03**	**NS**

Average values ± standard errors of ten independent samples are presented. Different letters in a row (a-b) represent statistically significant differences between treatments according to an LSD test at α < 0.05.

^A^Expressed in equivalents of caffeic acid.

^B^Expressed in equivalents of neochlorogenic acid.

The increased amount of light as a result of covering the orchard floor with reflective foil affected the content of gallic acid in persimmon flesh, which was 34% higher than in the control. This is consistent with the results reported by Feng *et al*.^23^. At the same time, they observed a significant difference in the content of caffeic acid between outer and shaded apple fruit flesh, which was not the case in our experiment. On the other hand, Andreotti *et al*.^24^ measured a significantly higher content of hydroxycinnamic acids in nectarine flesh in a reflective mulch treatment than in a control treatment. Other phenolics in the persimmon flesh did not change under the influence of reflective foil.

The content of flavan-3-ols (catechin) and flavonols (quercetin-3,4-diglucoside) in persimmon flesh did not change as a consequence of greater light reflection, which is partly in accordance with results reported by Andreotti *et al*.^24^.

Light quantity and quality affect phenolic compound synthesis in fruit, although the response is environmental and species dependent. In our experiment, the amount of light with reflective foil only increased by 5.6% compared to that of total solar radiation under the hail net and by 4% compared to that in the open field without a net. This may be why our results do not fully match all the previous reports listed above.

The phenolic compounds identified in our persimmon peel samples are listed in Table 5. The corresponding chromatogram on which identification was based is available as Supplementary Figs. S4 and S5. Among phenolic acids, an enhanced quantity of light increased the content of caffeoylquinic and gallic acids. Chlorogenic acid synthesis was shown to be only slightly light-regulated in an experiment by Jakopic *et al*.^25^ on ‘Fuji’ apples. A lower content of chlorogenic acid was measured in apple peels under a hail net, while its content was independent of the fruit position in the tree canopy. Similar results have been reported for ‘Jonagold’ apples in the Netherlands^26^. Cinnamic acids, in which caffeoylquinic and chlorogenic acid are classified, are reported to increase in ‘Stark Red Gold’ nectarine peels under the influence of reflective mulch^24^.

**Table 5:** Individual phenolic compounds and the sum of phenolic acids, flavan-3-ols and flavonols identified in persimmon peel and their content in the control and reflective foil treatment groups (mg kg^−1^ DW).

Individual phenolic compound	Control	Reflective foil	Significance
Gallic acid	20.9 ± 0.5 a	24.3 ± 1.0 b	*
Dihydrocaffeic acid 3-glucuronide^A^	28.0 ± 1.61	25.4 ± 1.1	NS
Caffeoylquinic acid^B^	25.2 ± 0.9 a	39.8 ± 1.7 b	*
**Phenolic acids**	**74.1 **±** 3.0 a**	**89.5 **±** 3.8 b**	*
Catechin	234.0 ± 7.1 a	297.2 ± 2.3 b	*
Procyanidin B1	285.4 ± 11.9 a	404.7 ± 12.3 b	*
Procyanidin dimer^C^	248.1 ± 9.8 a	350.8 ± 16.1 b	*
**Flavan-3-ols**	**767.5 **±** 28.8 a**	**1052.7 **±** 30.7 b**	*
Kaempferol-3-*O*-dihexoside^D^	16.7 ± 0.9 a	25.6 ± 1.2 b	*
Kaempferol-3-*O*-glucoside	102.3 ± 12.9	107.3 ± 6.5	NS
Kaempferol-3-*O*-galactoside^D^	40.0 ± 3.4 a	58.6 ± 2.0 b	*
Kaempferol-3-*O*-malonyl-hexoside^D^	10.0 ± 0.5	12.5 ± 1.2	NS
Kaempferol-3-*O*-(galloyl)-glucoside^4^	42.3 ± 4.9	47.3 ± 3.0	NS
Quercetin-3-*O*-glucoside	32.6 ± 2.7 a	47.1 ± 1.2 b	*
Quercetin-3-*O-*galactoside	28.9 ± 1.9 a	40.9 ± 0.8 b	*
Quercetin-3-*O*-rutinoside	8.5 ± 1.1	8.7 ± 1.0	NS
Quercetin*-*(galloyl)-hexoside^E^	89.3 ± 10.2	99.5 ± 6.3	NS
**Flavonols**	**370.6 **±** 38.5 a**	**447.5 **±** 23.2 b**	*

Average values ± standard errors of ten independent samples are presented. Different letters in a row (a-b) represent statistically significant differences between treatments according to an LSD test at α < 0.05.

^A^Expressed in equivalents of caffeic acid.

^B^Expressed in equivalents of neochlorogenic acid.

^C^Expressed in equivalents of procyanidin B1.

^D^Expressed in equivalents of kaempferol-3-*O-*glucoside.

^E^Expressed in equivalents of quercetin-3-*O-*rutinoside.

Unlike persimmon flesh, persimmon peels also contain procyanidin B1 and procyanidin dimer. The content of all identified flavan-3-ols in the persimmon peels significantly increased in fruits in the reflective foil treatment group compared with those observed in the fruit in the control group, which is consistent with the results reported by Andreotti *et al*.^24^ and Feng *et al*.^23^ (Table 5). Carbone *et al*.^27^ showed that in six strawberry cultivars, flavan-3-ol synthesis is more affected by genetic background than environmental factors, but they nevertheless observed some influence of the environment. Light is only one of many factors that alters enzymes involved in flavanol synthesis^27,28^. Surprisingly, other authors have shown that the synthesis of flavan-3-ols in different apple cultivars is not affected by light, and there were no significant differences in catechin content in apple fruits located in different parts of the canopy^25,26^. Chen *et al*.^29^ reported that the accumulation of individual phenolic compounds is significantly affected by both cultivar and shading treatment and, at the same time, their interaction, to which fruit species and environmental conditions can be added.

**Figure 2:**
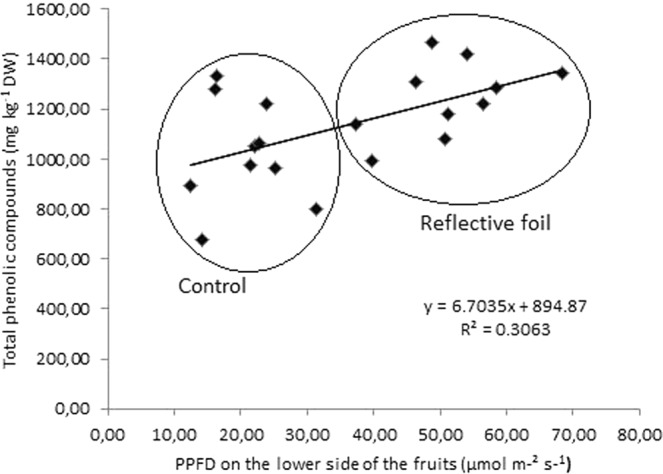
Relationship between total phenolic compound content (mg kg^−1^ DW) in persimmon peels and PPFD on the lower side of the fruits (µmol m^−2^ s^−1^) in the control and reflective foil treatment groups.

Flavonols identified in our persimmon peel samples were kaempferol and quercetin derivatives and are listed in Table 5. Persimmon peel contains significantly more flavonols than that in the flesh. The prevailing kaempferol derivative was kaempferol-3-*O*-glucoside, and the main derivative of quercetin was quercetin-(galloyl)-hexoside. The content of all individual flavonols in persimmon peel increased because of greater exposure to light in the reflective foil treatment, although not all differences were statistically significant. Other authors’ reports suggest that flavonol synthesis in apple and nectarine fruit peel is a light-dependent process because they measured a higher content of flavonols in fruits at the top and outer side of the canopy compared to that in fruits in the inner portion of the canopy^23–26^. Flavonol synthesis is generally more responsive to environmental conditions than to fruit developmental stage. Enhanced light quantity, especially shorter wavelengths, has been proven to increase flavonoid accumulation in fruits as a consequence of the increased activity of enzymes in the phenylpropanoid and flavonol biosynthesis pathways, such as phenylalanine ammonialyase (PAL) and flavonol synthase (FLS), respectively. The latter is the key enzyme for flavonol synthesis^27,30^. Reflective foil increased the quantity of light, reaching lower parts of persimmon fruits, which improved the total phenolic compound content in the persimmon peels (Fig. 2). The content of phenolics increases linearly with the amount of light (p = 0.01). However, the enhanced light quantity explains only 30.6% of the total phenolic compound content, while the other 69.4% remains unexplained with the experiment.

It can be concluded from our experiment that the accumulation of individual phenolic compounds, as a response to enhanced light quantity in the persimmon canopy, occurs differently in persimmon peel than in flesh. Ju *et al*.^13^ showed that genes ‘Fuji’ apples that are responsible for the synthesis of individual phenolic compounds react with different intensities to changes in light quantity. Many phenolics, such as chlorogenic acid, quercetin-3-glycoside and catechin, have the same biosynthetic pathway; however, their synthesis occurs independently from each other. This could explain our results. Awad *et al*.^26^ reported a similar conclusion in an experiment on ‘Fuji’ apples.

The fruit peel, which surrounds the fruit flesh, generally contains large amounts of bioactive compounds that have a beneficial effect on human health. Their content in fruit peel is significantly higher than in flesh, so persimmon peel should not be considered as waste but as part of an individual’s diet^31^.

## Supplementary information

Dataset 1
