# Supplementary material for: Influence of reflective foil on persimmon (Diospyros kaki Thunb.) fruit peel colour and selected bioactive compounds
Source: Sci Rep. 2019 Dec 13;9:19069. doi: 10.1038/s41598-019-55735-1 (PMC6911052; doi:10.1038/s41598-019-55735-1)
Supplement: Supplementary file 1 — Dataset 1 [file 41598_2019_55735_MOESM1_ESM.pdf]

Influence of reflective foil on persimmon (*Diospyros kaki* Thunb.) fruit peel colour and selected bioactive compounds

Tina Smrke,[1,]* Martina Persic,[1+] Robert Veberic,[1+] Helena Sircelj, [2+] Jerneja Jakopic[1+]

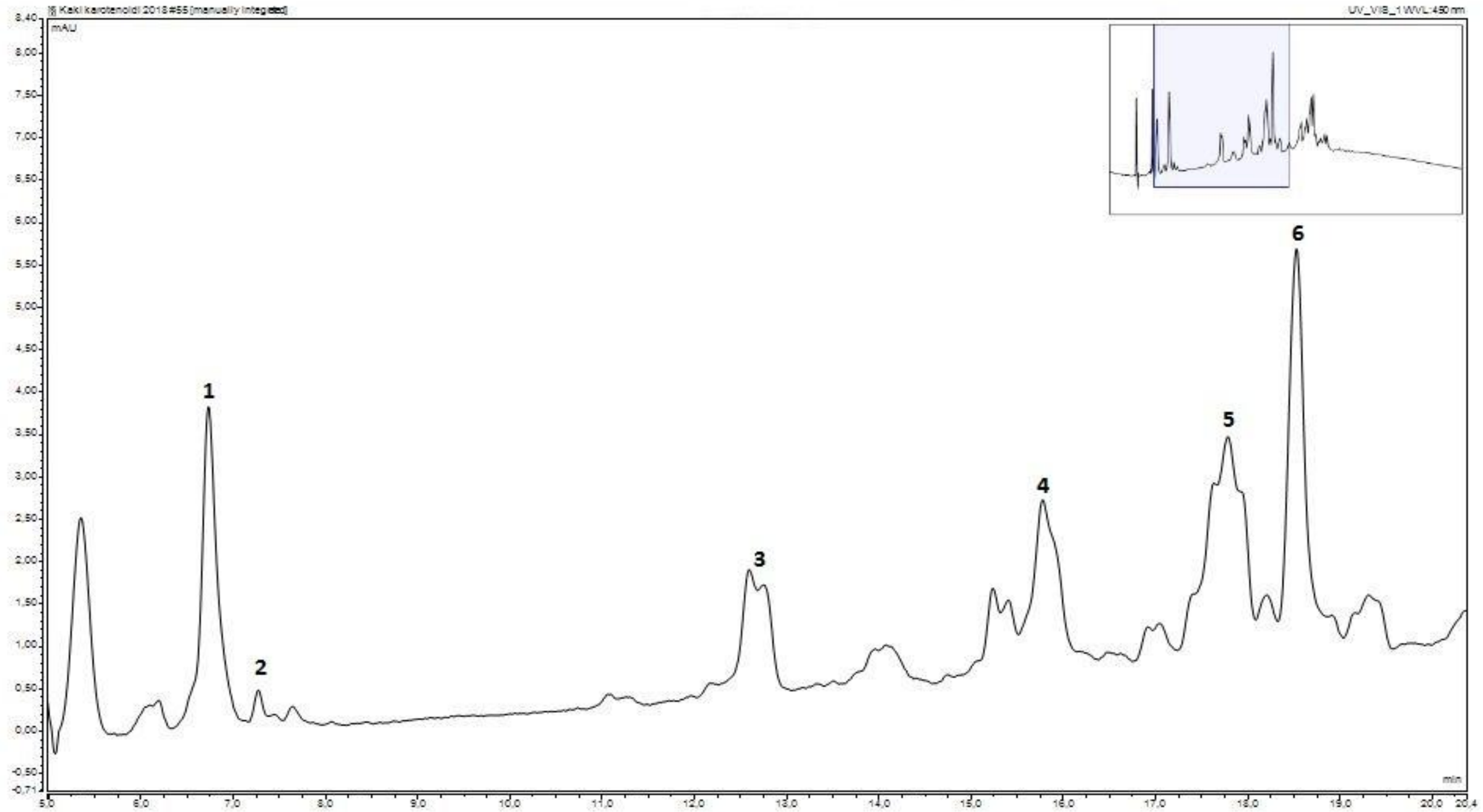

Supplementary Figure S1 Chromatogram for carotenoids at 450 nm of persimmon 'Tipo' fruit (**1** – lutein, **2-** zeaxanthine, **3** – β-cryptoxanthine, **4** – lycopene, **5** – α-carotene, **6** – β-carotene)

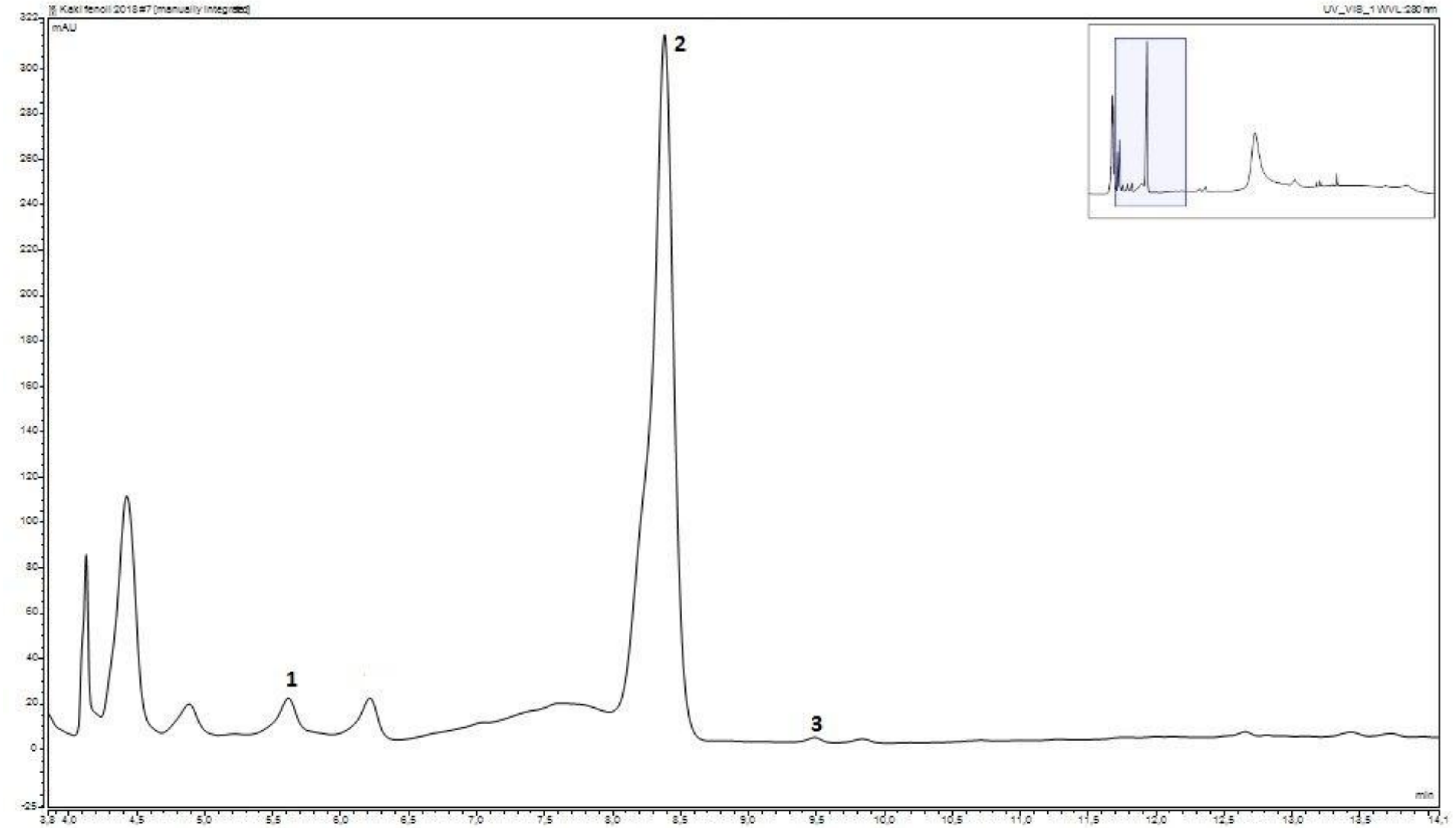

Supplementary Figure S2 Chromatogram for phenolic compounds at 280 nm of persimmon 'Tipo' flesh (**1** – gallic acid, **2**- dihydrocaffeic acid 3-glucuronide, **2** – caffeoylquinic acid, **3** – catechin)

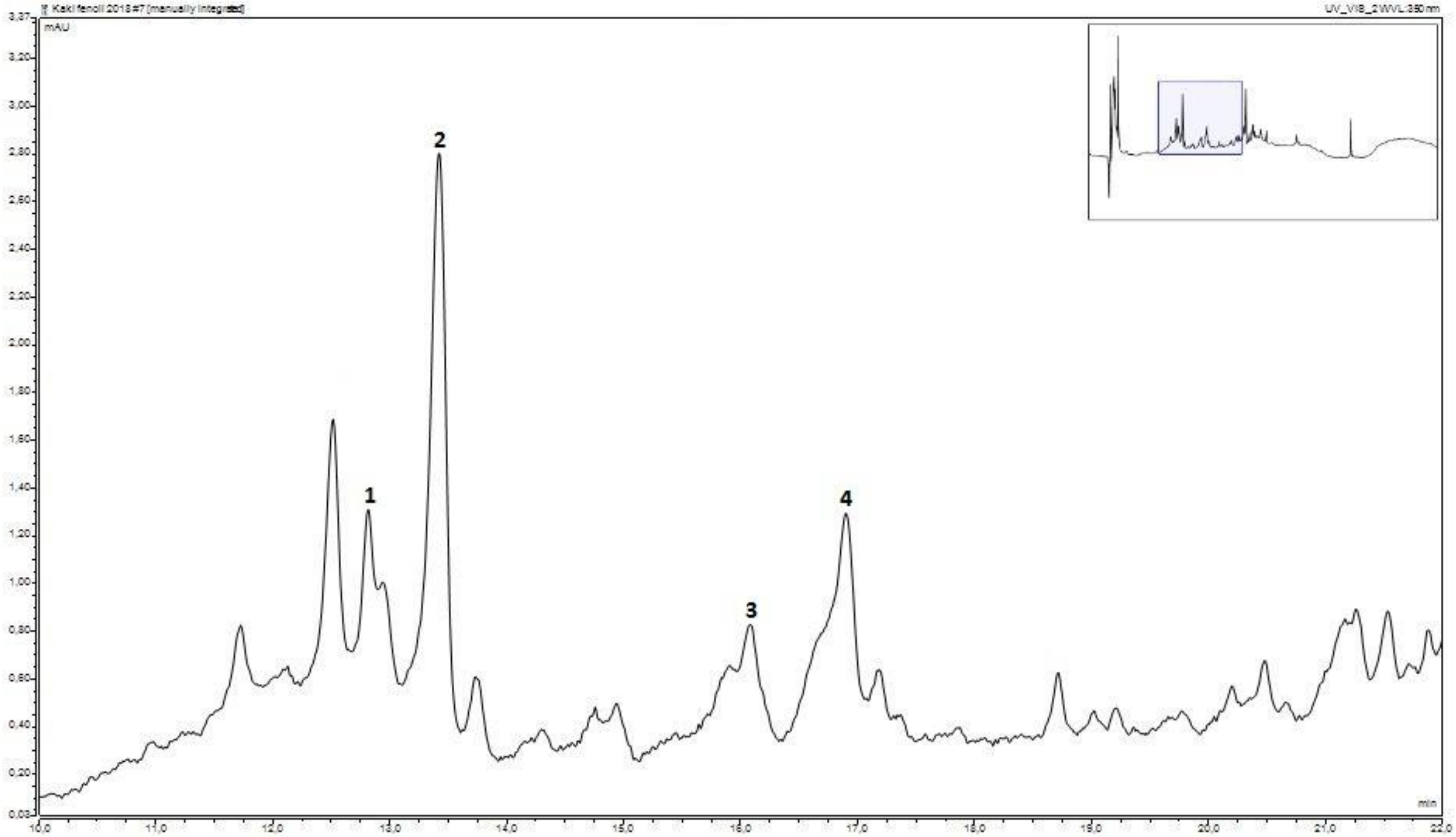

Supplementary Figure S3 Chromatogram for phenolic compounds at 350 nm of persimmon 'Tipo' flesh (**1** – Quercetin-3,4-diglucosid, **2**- caffeoylquinic acid, **3** – *p*-coumaric acid, **4** – *p*-coumaric acid)

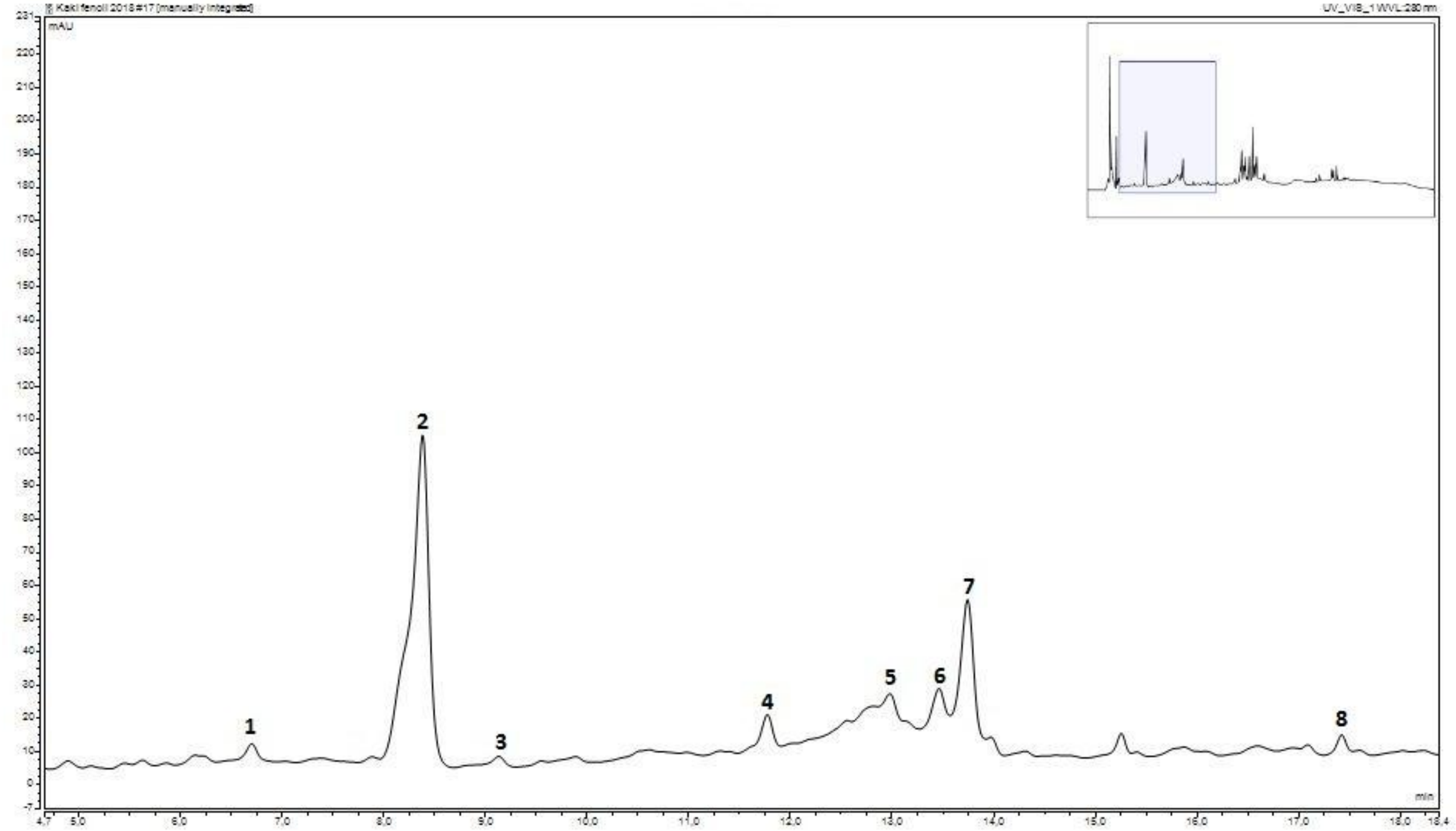

Supplementary Figure S4 Chromatogram for phenolic compounds at 280 nm of persimmon 'Tipo' peel (**1** – gallic acid, **2**- dihydrocaffeic acid 3-glucuronide, **3** – caffeoylquinic acid, **4** – procianidin B1, **5** – procianidin dimer, **6** – procianidin dimer, **7 –** catechin, **8** – procianidin dimer)

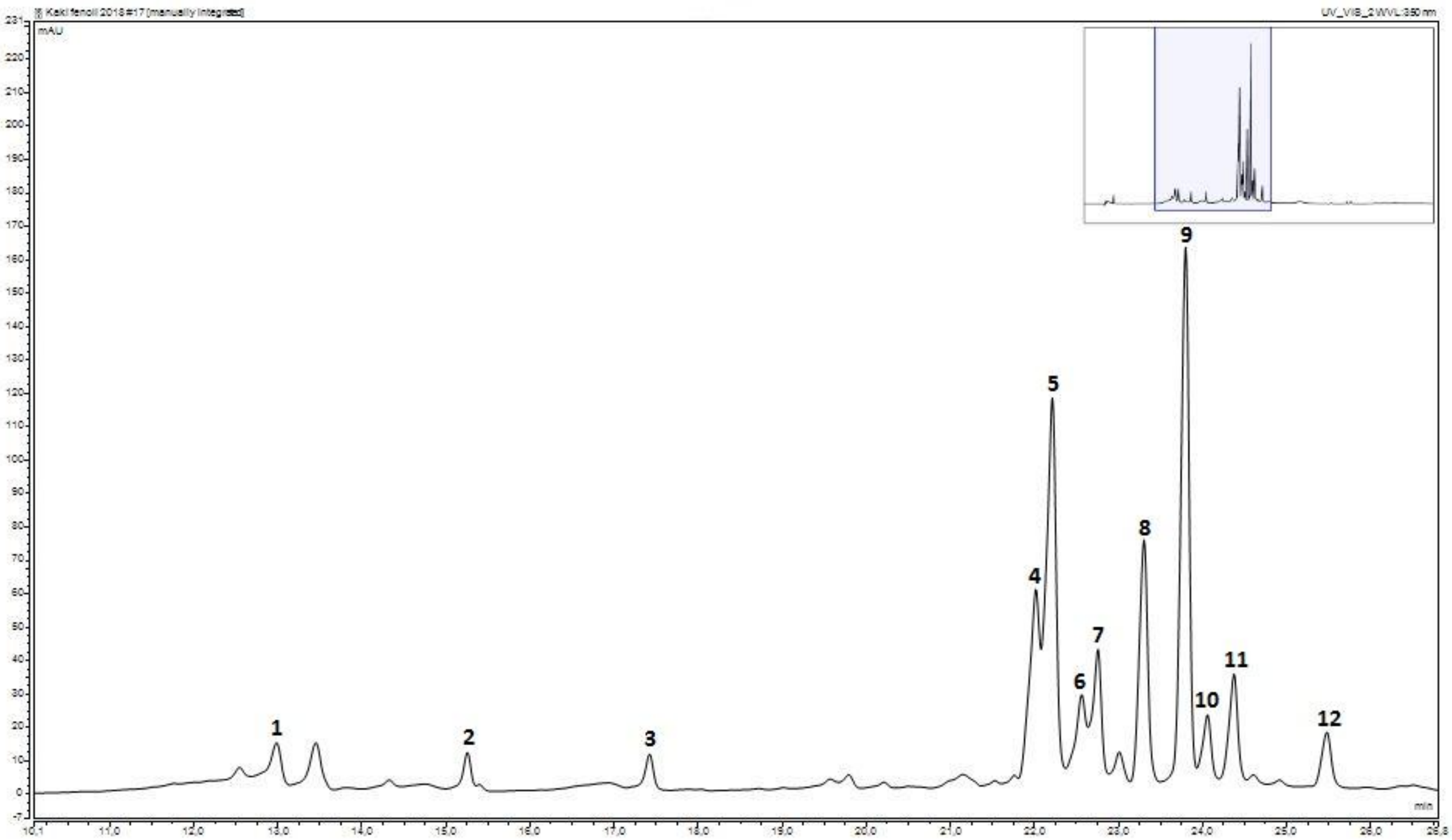

Supplementary Figure S5 Chromatogram for phenolic compounds at 350 nm of persimmon 'Tipo' peel (**1** – caffeoylquinic acid, **2**- kaempferol-3-*O*-dihexoside, **3** – quercetin-3-*O*-rutinoside, **4** – quercetin-3-*O*-galactoside, **5** – quercetin-3-*O*-glucoside, **6** – quercetin-(galloyl)-hexoside, **7 –** quercetin-(galloyl)-hexoside, **8** – kaempferol-3-*O*-galactoside, **9** – kaempferol-3-*O*-glucoside, **10** - kaempferol-3-*O*-(galloyl)-glucoside, **11** - kaempferol-3-*O*-(galloyl)-glucoside, **12** - kaempferol-3-*O*-malonyl-hexoside)
